# Analysis of reproductive outcomes after cesarean scar pregnancy surgery: a multicenter retrospective study

**DOI:** 10.3389/fmed.2025.1503836

**Published:** 2025-03-03

**Authors:** Yin Yin, Limei Huang, Nuo Xu, Huagang Ma, Chaoyan Yuan

**Affiliations:** ^1^Minda Hospital of Hubei Minzu University, Enshi City, Hubei, China; ^2^Linyi Municipal People’s Hospital, Linyi, Shandong, China; ^3^Henan Provincial People’s Hospital, Zhengzhou, Henan, China; ^4^Weifang Municipal People’s Hospital, Weifang, Shandong, China

**Keywords:** cesarean scar pregnancy, postoperative, recurrent cesarean scar pregnancy, reproductive outcomes, logistic regression

## Abstract

**Objective:**

This study aimed to analyze the outcome of postoperative re-pregnancies in patients with a cesarean scar pregnancy (CSP) and investigate the factors influencing the occurrence of recurrent cesarean section scar pregnancy (RCSP).

**Methods:**

A retrospective analysis was performed on the clinical data of 105 patients with CSP who had undergone surgical treatment and were admitted to the Minda Hospital affiliated with Hubei University for Nationalities, Henan Provincial People’s Hospital, Linyi People’s Hospital, and Weifang People’s Hospital from January 2015 to May 2021. The reproductive outcomes of these patients were monitored, and the factors influencing the occurrence of RCSP were analyzed.

**Results:**

In this study, it was found that the reproductive outcomes of patients with CSP after surgery included ectopic pregnancy, normal intrauterine pregnancy, RCSP, and abortion. The postoperative re-pregnancy rate was 51.72% (105/203), and the postoperative RCSP rate was 13.33% (14/105). The number of cesarean sections (OR = 2.004, 95% CI: 1.412–22.579, *p* < 0.001) was identified as an independent risk factor for the occurrence of RCSP, and the intraoperative removal of the uterine scar (OR = 0.045, 95% CI: 0.005–190.400, *p* = 0.002) was determined as an independent protective factor for the occurrence of RCSP.

**Conclusion:**

For patients with residual reproductive requirements after CSP surgery, the removal of uterine scar tissue during the operation can be contemplated. Subsequent postoperative re-pregnancy demands close surveillance and follow-up during gestation, with appropriate termination of pregnancy when warranted. For patients without reproductive needs after surgery, contraception is recommended to prevent the occurrence of RCSP.

## Introduction

Since the implementation of China’s “three-child policy,” the cesarean section rate among Chinese pregnant women has further increased (1, 2). Probably due to the increased frequency of cesarean sections, the incidence of cesarean scar pregnancy (CSP) in cesarean-section patients has also increased in recent years, with the incidence ranging from 1/1800 to 1/2200 (3). A variety of surgical treatments and medications are available for CSP, such as uterine artery embolization (UAE), methotrexate conservative drug therapy, curettage, hysteroscopic surgery, laparoscopic surgery, vaginal surgery, and open surgery. Studies on the application of methotrexate for conservative treatment of CSP have shown that the failure rates are as high as 25%, and a number of patients require subsequent surgical intervention (4, 5). Consequently, compared with the conservative treatment approach that solely utilizes drugs such as methotrexate, surgical treatment may be a preferable option for patients with CSP. Numerous studies have been conducted on the CSP surgical method; however, there are few studies on the reproductive outcomes of postoperative re-pregnancy in patients with CSP. Whether the variation in surgical methods influences the reproductive outcomes of postoperative re-pregnancy in patients with CSP remains to be explored. It is yet to be determined whether luteinizing hormone (LH), anti-Müllerian hormone (AMH), and follicle-stimulating hormone (FSH), which are commonly used as biological indicators for the evaluation of ovarian function, are correlated with the reproductive outcomes of postoperative re-pregnancy in patients with CSP (6, 7). In this study, we retrospectively analyzed the clinical data of 105 patients with postoperative re-pregnancy after CSP surgery, with the aim of exploring the outcomes of postoperative re-pregnancy in patients with CSP and the influencing factors for recurrent cesarean scar pregnancy (RCSP).

## Materials and methods

General information Between January 2015 and May 2021, a total of 1,204 patients with CSP who underwent surgical treatment were admitted to the Department of Obstetrics and Gynecology in the following hospitals: Hubei University for Nationalities Affiliated Minzu University Hospital, Henan Provincial People’s Hospital, Linyi Municipal People’s Hospital, and Weifang Municipal People’s Hospital. Among these patients, 296 received ultrasound-guided curettage, 402 underwent hysteroscopic surgery, and 502 had laparoscopic surgery. For all patients undergoing laparoscopic surgery, an ultrasonic knife was used to incise the muscle layer at the uterine isthmus mass for the removal of scar tissue. The uterine incision was closed in two layers in the double-layer arm using non-locking continuous multifilament sutures. The first layer included a significant portion of the myometrium and the endometrium. The second layer consisted of a continuous running suture that overlapped the first layer, incorporating the serosal layer and the superficial myometrial tissue. Among the 1,204 patients, 203 expressed the intention to conceive again after the operation. After screening based on the inclusion and exclusion criteria, a total of 105 patients with postoperative re-pregnancy following CSP were incorporated into this study.

Inclusion and exclusion criteria Inclusion criteria included the following: ① Patients who did not experience complications such as incisional infection or poor wound healing after surgery. ② Patients who expressed the desire to conceive again following surgery. ③ Patients who achieved their first natural pregnancy after the surgical procedure. ④ Patients who were planned to become pregnant again within the following specified time frames: Those who had undergone curettage or hysteroscopy were required to wait for at least 6 months, whereas those who had undergone laparoscopy were obligated to wait for a minimum of 1 year prior to attempting pregnancy again. Exclusion criteria included the following: ① Patients with a prior history of hysterectomy or tubal ligation. ② Patients with severe insufficiency of the liver, kidney, heart, or lung, or those with other severe underlying diseases. ③ Patients with incomplete medical record information. ④ Patients with interrupted follow-up records. This study obtained the approval of the Ethics Committee of our hospital, with the approval number V22017.

Follow-up For patients diagnosed with CSP who manifest the intention to conceive again, fasting venous blood samples will be procured on the second to fourth day of the first, third, and sixth menstrual cycles subsequent to surgery for the purpose of hormonal level evaluation. These patients will undergo follow-up assessments at 3-month intervals, with meticulous records being kept concerning their hormonal levels, pregnancy progression, perinatal conditions, and delivery particulars. In contrast, for patients with CSP who lack the intention to conceive again, a solitary follow-up will be implemented in the third month post-surgery, during which no hormonal level assay will be performed. Subsequently, the patients were stratified into the intrauterine pregnancy group (*N* = 91) and the RCSP group (*N* = 14) in accordance with their postoperative re-pregnancy outcomes, with the objective of probing into the postoperative re-pregnancy and delivery states among CSP patients as well as the determinants underlying the occurrence of RCSP.

CSP typing method In 2016, the Family Planning Group of the Obstetrics and Gynecology Section of the Chinese Medical Association published the “Expert Consensus on the Diagnosis and Treatment of Uterine Keloid Pregnancy after Cesarean Section” (2016) (8). In the consensus, the clinical classification of CSP was detailed: Type I: The pregnancy tissue is partially implanted in the uterine scar, partially or mostly located in the uterine cavity, and a few reach the base of the uterus. The myometrium between the pregnancy tissue and the bladder becomes thin, and the thickness is >3 mm. Type II: A part of the pregnancy tissue is implanted in the uterine scar, part or most of it is located in the uterine cavity, and a few reach the bottom of the uterine cavity. The uterine muscle layer between the pregnancy tissue and the bladder becomes thin, and the thickness is <3 mm. Type III: The pregnancy tissue is completely implanted in the muscle layer of the uterine scar and protrudes outward toward the bladder. The uterine cavity and cervical canal are empty. The myometrium between the pregnancy tissue and the bladder is thin or missing, and the thickness is <3 mm.

### Statistical analysis

Statistical analysis was performed using SPSS version 26.0 software and SPSSAU software. Continuous variables conforming to normal distribution were expressed as mean ± standard deviation ( $\bar{x}$ ± s), and one-way ANOVA was used for comparison between groups. The Box-Cox transformation is applied to the raw data of the non-normal distribution to make it appear to be normally distributed, so as to facilitate subsequent data processing and provide more accurate data for subsequent research. Quantitative data should be described by percentage, and the *X*^2^ test was used for comparisons between groups using the *X*-test, with *α* = 0.05 as the test level and *p* < 0.05 as the statistically significant difference. Influential factors that were statistically significant in the univariate analysis were included in the binary logistic regression model for analysis, and a *p* < 0.05 was taken as the statistically significant difference.

## Results

General data No statistically significant differences were detected between the two groups in terms of age, gravidity, the interval from the last cesarean section, the diameter of the CSP gestational sac, the gestational age at CSP diagnosis, the preoperative blood *β*-HCG level, the postoperative blood β-HCG recovery period, the postoperative menstrual recurrence time, the incidence of reduced postoperative menstrual flow, and the CSP type (*p* > 0.05). The mean number of previous cesarean sections was markedly elevated in the RCSP group (3.58 ± 1.38) in contrast to that in the intrauterine pregnancy group (2.89 ± 1.07), exhibiting a statistically significant difference (*p* < 0.05). The intraoperative uterine scar excision rate was 14.29% (2/14) for the RCSP group, which was conspicuously lower than that of the intrauterine pregnancy group (88.17%, 82/91), manifesting a statistically significant difference (*p* < 0.05) (refer to Table 1 for detailed data).

**Table 1:** General information about patients in both groups.

	RCSP group (*N* = 14)	Intrauterine pregnancy group (*N* = 91)	*t/X* ^2^	*P*
Age (years)	31.29 ± 5.26	32.20 ± 4.70	1.963	0.056
Number of pregnancies	3.58 ± 1.38	3.29 ± 1.16	1.677	0.469
Number of previous cesarean sections	3.58 ± 1.38	2.89 ± 1.07	1.074	0.046
Time since the last cesarean section (years)	4.73 ± 0.92	5.06 ± 1.05	1.782	0.309
CSP gestational sac diameter (cm)	3.26 ± 0.22	3.73 ± 00.45	2.544	0.199
CSP gestational age (days)	44.77 ± 5.05	42.39 ± 4.93	1.432	0.603
Preoperative blood β-HCG (mIU/L)	25776.23 ± 872.57	28923.49 ± 519.62	4.906	0.112
Time of blood β-HCG returning to normal (days)	32.68 ± 4.02	33.02 ± 3.38	2.562	1.020
Recovery time of menstruation (days)	31.71 ± 3.01	30.15 ± 2.79	1.472	0.621
Decrease in postoperative menstrual flow (%)	7.14% (2/14)	5.49% (5/91)	2.051	0.080
CSP type				
CSP I	35.71% (5/14)	41.76% (38/91)	0.871	0.082
CSP II	35.71% (5/14)	35.16% (32/91)		
CSP III	28.58% (4/14)	23.08% (21/91)		
Intraoperative removal of uterine scar (%)	14.29% (2/14)	88.17% (82/91)	8.354	<0.001

Postoperative hormone levels of patients in the two groups No statistically significant differences were detected in the comparisons of the three hormone levels, namely, FSH, LH, and AMH, between the two groups of patients prior to surgery, as well as 1 month, 3 months, and 6 months after surgery (*p* > 0.05). When comparisons were made within each group of patients, no statistically significant differences were found between the hormone levels at 1 month, 3 months, and 6 months after the operation and those before the operation (*p* > 0.05) (refer to Table 2 for detailed information).

**Table 2:** Postoperative hormone levels in two groups of patients.

		RCSP group (*N* = 14)	Intrauterine pregnancy group (*N* = 91)	*t/X* ^2^	*P*
LH (mIU/ml)	Preoperative	6.83 ± 3.52	7.02 ± 3.79	1.712	0.883
	1 month after surgery	5.97 ± 2.74	6.52 ± 2.34	2.081	0.625
	3 months after surgery	6.33 ± 3.01	6.69 ± 2.75	1.901	0.741
	6 months after surgery	6.92 ± 2.88	7.05 ± 1.44	1.375	1.211
	*P*	>0.05	>0.05		
FSH (mIU/ml)	Preoperative	7.81 ± 1.11	8.12 ± 1.74	2.057	0.654
	1 month after surgery	6.85 ± 2.33	7.66 ± 2.15	1.617	0.712
	3 months after surgery	7.24 ± 1.79	7.85 ± 2.31	3.220	0.182
	6 months after surgery	7.91 ± 1.97	8.20 ± 2.41	1.237	0.249
	*P*	>0.05	>0.05		
AMH (ng/ml)	Preoperative	3.33 ± 0.52	3.84 ± 0.96	0.964	0.931
	1 month after surgery	3.26 ± 1.18	3.66 ± 0.74	2.005	1.032
	3 months after surgery	3.47 ± 0.79	3.86 ± 1.02	2.001	0.090
	6 months after surgery	3.55 ± 0.83	3.92 ± 0.83	1.390	0.170
	*P*	>0.05	>0.05		

**Figure 1:**
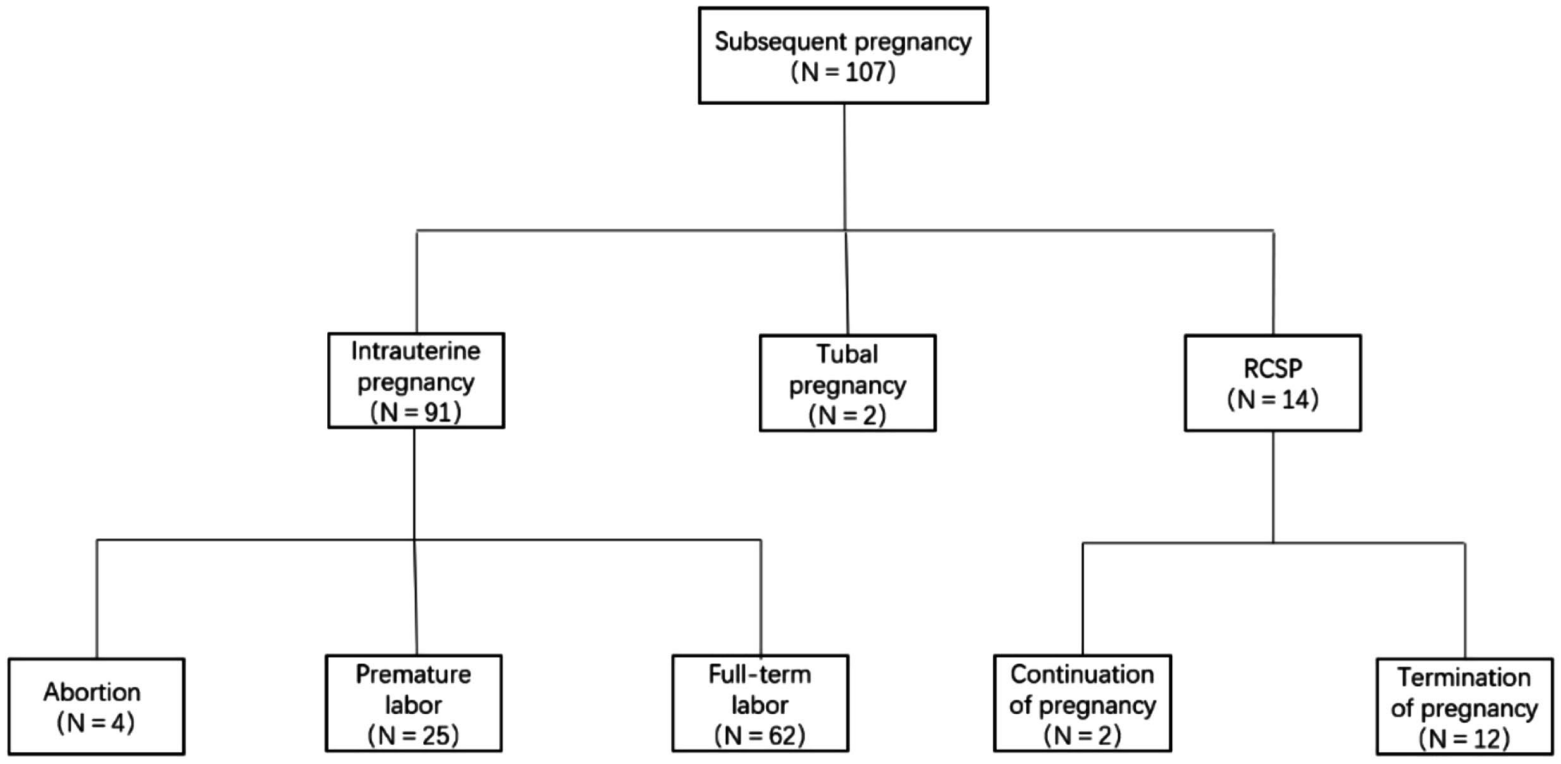
Outcome of re-pregnancy after CSP surgery.

Perinatal situation in the intrauterine pregnancy group Among the 91 patients experiencing an intrauterine pregnancy, two cases of missed miscarriage, two cases where patients voluntarily terminated the pregnancy due to personal reasons, and 25 cases of preterm births via cesarean section were observed, yielding a preterm labor rate of 27.48% (25/91). The etiologies of preterm delivery encompassed the following: Ultrasonography indicated that the thickness of the thinnest part of the myometrium in the lower uterine segment was <0.1 mm in four patients, premature rupture of membranes and oligohydramnios were present in two patients, placenta accreta was noted in eight patients, twin gestation occurred in two patients, a combination of gestational diabetes or hypertensive disorders in pregnancy was diagnosed in four patients, complete placenta previa was identified in two patients, and fetal distress was detected in three patients. The aforementioned preterm neonates had a gestational age ranging from 28 to 36 weeks, and all survived subsequent to rescue treatment in the neonatal intensive care unit (NICU). Among the 91 patients with intrauterine pregnancy, 62 patients underwent cesarean section at full-term gestation. The vast majority of these patients did not experience any conspicuous complications or placenta-related disorders during pregnancy. Ultimately, all of them were delivered via cesarean section, with the operations proceeding smoothly and resulting in healthy newborns, yielding a full-term delivery rate of 68.13% (62/91) (refer to Figure 1 for a detailed visual representation).

Perinatal situation in the RCSP group. The incidence rate of recurrent cesarean scar pregnancy (RCSP) in the present study was determined to be 13.33% (14/105). Among the 14 patients diagnosed with RCSP, 12 opted for pregnancy termination immediately upon the detection of RCSP during early gestation, while 2 elected to continue with the pregnancy. Among the two patients with RCSP who elected to continue the pregnancy, one was diagnosed with type II CSP via ultrasonography at 7 weeks of gestation. Despite being informed by the physician of the potential risks associated with persisting in the pregnancy, the patient chose to carry on. At 18 weeks of gestation, ultrasonography revealed placenta previa and placenta accreta, prompting a recommendation for pregnancy termination. Nevertheless, the patient persisted in her decision to continue her pregnancy. At 23 weeks of gestation, ultrasonography demonstrated placenta previa, placenta accreta, and cervical dilatation. Subsequently, the patient elected to discontinue the pregnancy and underwent a cesarean section, during which a diagnosis of placenta increta was established, accompanied by an intraoperative blood loss of approximately 1,100 mL. Fortunately, the uterus was conserved during the surgical procedure. The other patient with RCSP who opted to continue the pregnancy was classified as having type I CSP. At 35 weeks of gestation, due to the onset of regular contractions and a progressively shortening cervical canal, a cesarean section was performed for pregnancy termination.

Analysis of the influencing factors for the occurrence of RCSP. The influencing factors that demonstrated statistical significance in the aforementioned analysis were subjected to logistic regression analysis. The results indicated that the number of cesarean deliveries [odds ratio (OR) = 2.036, 95% confidence interval (CI): 1.457 to 22.873, *p* < 0.001] emerged as an independent risk factor for the occurrence of recurrent cesarean scar pregnancy (RCSP), whereas intraoperative removal of the uterine scar (OR = 0.045, 95% CI: 0.005 to 190.400, *p* = 0.002) served as an independent protective factor against the occurrence of RCSP (refer to Table 3 for detailed data).

**Table 3:** Logistic regression analysis of factors influencing postoperative recurrence of CSP.

Variables	*β*	SE	Wald	*P*	OR	95% CI
Number of cesarean sections	1.461	0.333	16.933	<0.001	2.044	1.412–22.579
Intraoperative removal of uterine scar	−3.092	0.975	10.083	0.002	0.045	0.005–190.400

## Discussion

In the present study, the perioperative levels of LH, FSH, and AMH among the two groups of patients were analyzed. It was found that no statistically significant differences were observed, whether when comparing the hormone levels of different groups of patients during the same period or when comparing those of patients in different periods within the same group. These findings demonstrate two aspects: First, there is no significant difference in the levels of LH, FSH, and AMH between patients with normal intrauterine pregnancy and those with RCSP. Second, curettage, hysteroscopy, and laparoscopy, which are currently the common surgical approaches for terminating pregnancy in CSP, do not exert a significant impact on the levels of LH, FSH, and AMH among patients in the postoperative period.

A meta-analysis conducted by Morlando et al. (9) demonstrated that the rate of live births at term among women possessing a history of CSP and becoming pregnant subsequent to surgical treatment was 54.9%. The live birth rate within the intrauterine pregnancy group in the current study was 68.13% (62/91), which exceeded that in the research by Morlando et al. (9). The possible explanation for this disparity might be attributed to the fact that the study by Morlando et al. (9) incorporated a substantial proportion of women with unplanned pregnancies who lacked regular prenatal examinations. Consequently, it is of great significance for women who conceive again after CSP to undergo regular prenatal checkups and receive perinatal care under the guidance of a physician as this might enhance the live birth rate.

In our study, a patient diagnosed with CSP type I sustained her pregnancy until the 35th gestational week and achieved a successful live infant delivery via cesarean section. Moreover, a patient with CSP type II terminated her pregnancy during the second trimester due to placenta implantation. CSP patients who underwent expectant treatment display a high degree of variability in pregnancy outcomes, which consequently renders the crucial concern of how to identify appropriate patients at an early stage. Fang et al. (10) investigated 11 CSP patients under expectant management. Their research findings indicated that among the nine cases in the advanced pregnancy phase, seven were classified as CSP type I and two as CSP type II; furthermore, the two instances of uterine rupture during the second trimester were both of CSP type III. Subsequently, certain scholars suggest that pregnant women diagnosed with CSP type I or II who possess a strong determination to continue their pregnancy, following a comprehensive understanding of the associated risks, may proceed with their pregnancy under strict surveillance within a tertiary medical institution (11, 12). However, the reason why the CSP II patients in this study underwent placental implantation in the second trimester and failed to sustain the pregnancy successfully remains to be elucidated. This phenomenon might be associated with the frequency of CSPs that the patient has encountered. A comparative analysis of the studies conducted by Calì et al. (13) and Fang et al. (10) with respect to the maintenance of pregnancy in CSP patients demonstrates that the subjects in their respective studies were all patients with an initial occurrence of CSP. In contrast, the CSP patients in the current study who sustained their pregnancies were all RCSP patients. It is hypothesized that the augmented frequency of CSPs that the patient has endured may attenuate the prognostic value of CSP classification in relation to the sustenance of a secure pregnancy among CSP patients.

Tantbirojn et al. (14) reported an incidence rate of 25% for RCSP. Wang et al. (15) documented a 15.6% incidence rate of RCSP in their study (5). Maymon et al. (16) conducted an analysis of the clinical data of 90 patients with CSP, revealing a postoperative pregnancy rate of 48% (43/90) and an RCSP rate of 11% (10/90). In our study, it was found that the postoperative outcomes of re-pregnancy among patients with cesarean scar pregnancy (CSP) encompassed ectopic pregnancy, normal intrauterine pregnancy, recurrent cesarean scar pregnancy (RCSP), and abortion. The postoperative pregnancy rate was determined to be 51.72% (105/203), and the postoperative RCSP rate was 13.33% (14/105), which was generally consistent with the findings of the aforementioned studies. In the present study, among the patients in the intrauterine pregnancy group, placental implantation was identified as the most prevalent complication during pregnancy, with an incidence rate of 8.80% (8/91). The possible reason for this could be analyzed as follows. Cesarean scar pregnancy (CSP) and placental implantation share a similar pathogenesis (17, 18), which is associated with uterine decidual injury and abnormal invasion of chorionic trophoblast cells in the lower segment of the scarred uterus (19–23). Consequently, the incidence of placental implantation is elevated in the second pregnancies of patients with a prior history of CSP.

In the current study, it was ascertained that the number of cesarean deliveries (OR = 2.044, 95% CI: 1.412 to 22.579, *p* < 0.001) constituted an independent risk factor for the development of RCSP, whereas intraoperative removal of the uterine scar (OR = 0.045, 95% CI: 0.005 to 190.400, *p* = 0.002) served as an independent protective factor for the development of RCSP. The necessity of removing and repairing the uterine scar resulting from cesarean delivery remains a subject of debate. Whether such repair can alter reproductive outcomes is a contentious issue. Several studies have demonstrated that excision of the uterine scar accompanied by repair of the muscular tissue can diminish the incidence of RCSP (14–17). As previously expounded, the existence of uterine scar tissue is likely to augment the probability of uterine decidual injury and abnormal invasion of chorionic trophoblast cells within the lower segment of the scarred uterus, thereby giving rise to a comparatively higher incidence of CSP and placental implantation. Furthermore, existing research has indicated that there exists a negative correlation between the thickness of the myometrium of the anterior wall of the lower uterine segment and the number of cesarean deliveries. Specifically, an increment in the number of cesarean deliveries correlates with a reduction in the thickness of the myometrium of the anterior wall of the lower uterine segment, concomitantly elevating the risk of uterine rupture during mid to late pregnancy (24). Consequently, from the vantage point of curtailing the incidence of RCSP and averting uterine rupture during pregnancy, it may be requisite to excise the uterine scar in patients with CSP. However, it has also been posited that routine performance of uterine scar excision and myometrial repair surgery in such patients would culminate in augmented surgical complexity, elongated surgical duration, and elevated surgical costs (25). In the present study, it was discerned that intraoperative excision of the uterine scar functions as an independent protective factor against the development of RCSP. Given that the myometrium at the scar on the anterior wall of the uterus is characteristically thin and replete with blood supply, we posit that scar excision is a viable option for patients desiring subsequent childbearing.

In conclusion, the potential outcomes of pregnancy subsequent to CSP treatment encompass ectopic pregnancy, spontaneous abortion, RCSP, and normal intrauterine pregnancy. These pregnancies may be complicated by placental implantation, placenta previa, and uterine rupture during the middle and late stages of gestation. Moreover, the majority of such pregnancies are likely to be terminated via elective cesarean section. Early detection and prompt treatment of RCSP can mitigate the occurrence of serious complications. Hence, for patients with reproductive requirements following CSP treatment, appropriate therapeutic strategies ought to be selected in light of the patient’s individual circumstances. During pregnancy, close monitoring and follow-up of the pregnancy progress are imperative, and the pregnancy should be terminated at an opportune time if necessary. For patients without reproductive intentions after treatment, it is advisable to adopt safe, long-term, and reliable contraceptive measures to preclude the recurrence of CSP.

However, it should be noted that the four hospitals incorporated into this study are all large tertiary care institutions. The pronounced trust that Chinese patients with CSP place in major hospitals, in conjunction with the possible presence of subjective selection bias, might result in the incidence rate of CSP in these four hospitals surpassing the range of 1/1800 to 1/2200. In addition, this study was constrained by its retrospective nature and dependence on funding sourced from policy initiatives. Specifically, no data were collected regarding the estradiol (E2) hormone levels in patients with CSP, nor were data obtained on the hormone levels of those patients with CSP who had no intention of conceiving again after surgery. It is anticipated that in subsequent prospective studies, these limitations will be tackled by incorporating hospitals of diverse levels, augmenting the heterogeneity of the patient cohort, and instituting a comprehensive data collection protocol for patient hormone levels.

## Data Availability

The raw data supporting the conclusions of this article will be made available by the authors, without undue reservation.
